# Empowering yet challenging: managing multiple sclerosis in the workplace—an interview study

**DOI:** 10.3389/fpubh.2026.1643923

**Published:** 2026-01-22

**Authors:** Jessica Dervish, Veronica Svärd, Kyla A. McKay, Alejandra Machado, Agneta Wennman-Larsen, Emilie Friberg

**Affiliations:** 1 The Division of Insurance Medicine, Department of Clinical Neuroscience, Karolinska Institutet, Stockholm, Sweden; 2 Department of Social Work, Södertörn University, Huddinge, Sweden; 3 Neuro Division, Department of Clinical Neuroscience, Karolinska Institutet, Stockholm, Sweden; 4 Center for Molecular Medicine, Karolinska Hospital, Stockholm, Sweden; 5 Department of Nursing Science, Sophiahemmet University, Stockholm, Sweden

**Keywords:** chronic disease, concealment, disclosure, multiple sclerosis, self-management, strategies, worklife, workplace

## Abstract

**Background:**

Having a chronic disease such as multiple sclerosis (MS) may pose challenges for affected individuals in their work life. Given its progressive nature and wide range of symptoms, living with MS requires ongoing adaptation. In addition to work accommodations and support, individuals may develop their own strategies to manage their condition at work.

**Objectives:**

To explore experiences of people with MS (PwMS) in managing MS in the workplace, focusing on strategies they employ to facilitate their work lives and challenges they navigate.

**Methods:**

Semi-structured individual interviews were held with 16 working PwMS in Sweden. Interviews were analyzed using reflexive thematic analysis.

**Results:**

Three main themes were identified that highlight strategies for managing MS. The theme “Adapting to MS”, included statements on how self-reflection led to strategies more aligned with health needs and understanding the best practices and pitfalls of managing a chronic disease. “Taking responsibility in the workplace” described the participants’ commitment to protect their health at work, and efforts made to create an environment more conducive to MS, and last, “Approaching sustainability” included reports on making sustainable choices in relation to work life.

**Conclusion:**

This study provides insights into the experiences of those living and working with MS. The results demonstrate the active agency in managing their condition. PwMS employed various strategies; concentrating on adapting to MS mentally and practically, taking an active stance for their health, and making choices based not only on their current health status but also on potential future circumstances.

## 1 Introduction

Multiple sclerosis (MS) is a chronic neurological disease characterized by periods of relapses and remissions (1), with onset typically occurring in adults of working age (2). Most people with MS (PwMS) eventually experience a steadily progressive course, marked by a gradual decline in overall health and functioning. While there is currently no cure for MS, increasingly effective treatments (3, 4) have become available, which minimize disease activity and the frequency of relapses. Nonetheless, work participation for PwMS can be challenging, especially during relapses (5, 6).

PwMS often experience a mixture of visible and invisible symptoms (7, 8), which can be highly unpredictable and often disabling. Fatigue, mobility impairments, and cognitive deficits are particularly challenging and can greatly affect work-related activities (9). As MS symptoms can vary widely in intensity and impact from day to day, the disease requires continual adaptation, which may add an extra layer of complexity in dealing with professional responsibilities. Previous research shows how work difficulties, stigma, and reduced quality of life are prevalent even among PwMS with low physical disability (10), demonstrating that challenges are not limited to late-stage physical impairment.

While work adjustments and the attitudes of employers and colleagues are often essential for creating a more sustainable work situation among PwMS, individuals develop their own strategies to facilitate their work lives in light of their condition. This can be described as a form of self-management (11–13). For this study, the definition by Barlow et al. was adopted: “an individual’s ability to manage the symptoms, treatment, physical and psychological effects, social implications, and lifestyle changes associated with living with a chronic condition.” They further emphasize that self-management refers to the ability to manage one’s health condition and, through cognitive, behavioral, and emotional responses, strive to maintain a good quality of life (14). Studies suggest that, in general, practical strategies for self-management often involve learning pacing techniques as well as maintaining energy levels (15, 16) and using aids to prevent physical strain (17, 18). On a behavioral level, strategies may include improved prioritization (19), and continuously learning about the condition and identifying health needs as they arise (20).

We further draw on two additional frameworks: (1) Strauss and colleagues’ *“illness work”* framework (21, 22), which describes the substantial yet often hidden work involved in managing a chronic illness; and (2) Bury’s (23) notion of *“biographical disruption”*, which conceptualizes chronic illness as a disruptive event that challenges taken-for-granted assumptions and prompts individuals to reassess their life trajectory and sense of self.

Although research focusing specifically on MS self-management in workplace contexts is emerging (24–26), most existing literature addresses broader employment experiences (27), workplace adjustments, work-related challenges (28), and self-reported work productivity (29). However, little is known about the everyday strategies PwMS use to manage their condition at work and the role that workplace structures can play in shaping these strategies (24). This is important because self-management often emphasizes individual responsibility (14), yet in workplace settings, individuals must navigate organizational norms, resources, and the complex decision whether to disclose their condition; a choice that can significantly impact their health, day-to-day interactions, and work life (30). Research focusing on this topic in Swedish workplaces is particularly scarce. Given Sweden’s strong employment protections (31), it offers an interesting context for examining self-management in relation to workplace dynamics. Consequently, the aim of this study was to explore the experiences of PwMS as they manage MS in the workplace, focusing on the strategies they employ to facilitate their work lives and the challenges they navigate.

Addressing this gap is crucial for developing more effective workplace policies and interventions that can support PwMS in balancing their health needs with their professional responsibilities.

## 2 Methods

Semi-structured interviews were used to gain an in-depth understanding of the study topic. An interview guide was developed so that the interviewer (JD) could be flexible to the participant’s perspectives, making it possible to obtain a wide range of experiences. The interview guide consisted of three broad themes: (1) strategies to manage MS in relation to work; (2) experiences of disclosure and concealment of MS in the workplace; and (3) reflections on past choices in relation to their work and health. It also included introductory questions regarding age, current occupation, time of MS diagnosis, and how the disease first presented, to give context to their experiences.

Inclusion criteria for participants in the study were:

- A formal diagnosis of MS
- Currently or recently working at least half-time
- Over 18 years of age
- Able to understand and communicate fluently in Swedish
- Able to provide written informed consent

### 2.1 Recruitment and data collection

PwMS were invited to register their interest in participating in the study through the website of a Swedish MS patient organization. The main author, JD, then reached out to these individuals (23 in total) via email and provided detailed information about the study to confirm their continued interest in participating. One reminder was sent. An additional participant was recruited through snowball sampling. A total of 16 PwMS participated in the study. Even if data saturation was reached before the final interviews, JD continued with all scheduled participants as they had already volunteered to participate (see Table 1).

**Table 1:** Demographic details of the participants.

Pseudonym	Sex	Age	Time since diagnosis	Employment status	Disclosure status in current or last held job	Type of work (office/manual)
Emil	Man	35	2 years	Full-time	Partially disclosed	Manual
Sophie	Woman	42	1 year	Full-time	Fully disclosed	Office
Nils	Man	36	7 years	Full-time	Fully disclosed	Office
Klara	Woman	39	2 years	Full-time	Partially disclosed	Office
Martin	Man	40	16 years	Full-time	Partially disclosed	Office
Annelie	Woman	44	6 years	Full-time	Partially disclosed	Office
Kristian	Man	42	3 years	Full-time	Fully concealed	Office
Elsa	Woman	25	6 months	Full-time	Fully disclosed	Office
Malin	Woman	49	11 years	Full-time	Fully concealed	Office
Jesper	Man	35	1 year	Full-time	Fully disclosed	Office
Marie	Woman	51	5 years	Part-time (75%)	Fully disclosed	Manual
Anton	Man	34	3 years	Full-time	Fully disclosed	Manual
Louise	Woman	41	18 years	Half-time (50%)	Fully concealed	Office
Tobias	Man	57	8 years	On disability pension	Fully disclosed	Office
Linda	Woman	25	6 years	Part-time (65%)	Partially disclosed	Manual
Elisabet	Woman	29	12 years	Full-time	Partially disclosed	Office

JD, who has previous experience interviewing for qualitative research purposes, conducted the interviews. JD had no prior relationship with, or knowledge of, any of the participants. Recruitment and interviews were conducted between August 2023 and March 2024. Before each interview began, all participants signed a consent form that confirmed their voluntary participation in the study.

The interviews were conducted individually, at a time and place that was convenient for each participant, either in person (*n* = 1), by video (*n* = 12), or phone (*n* = 3), depending on their preferences. Interviews took place on one occasion and lasted approximately 45–60 min. The interviews were conducted in Swedish, audio-recorded, and transcribed verbatim by JD. To ensure confidentiality, all sensitive information was de-identified during the transcription process.

### 2.2 Data analysis

The gathered data were analyzed using a reflexive thematic analysis (RTA) approach (32). This method recognizes that researchers’ preconceptions inevitably influence their interpretations and encourages transparency about the context of these interpretations and the experiences being studied (32, 33). To enhance transparency in the research process, field notes were written immediately after each interview by JD. These notes served as a research journal, documenting reflections on both the interview content and the interaction with participants, and offered descriptive and analytical insights that informed initial coding and early theme development. Transparency was further ensured by keeping an audit trail: documenting each step of the research process.

JD conducted the analysis, in continuous collaboration and discussions with EF, AWL, and VS, in the following six steps:

1. *Familiarizing with dataset.* The interview transcripts were repeatedly read by JD to familiarize herself with and gain a deep grasp of the material before the coding began. AWL, EF, and VS also read a selection of different interviews to familiarize themselves with the data.
2. *Coding.* All quotes relevant to the research aim were extracted and organized into meaningful groups by JD. The quotes were read several times to identify concepts, similarities, and differences. Features that were identified as important were then condensed and assigned a code. In this step, information that might have been important for the participants’ overall self-management, but not particular to the workplace, was noted but not included in the coding.
3. *Generating initial themes.* Related codes were grouped into broader themes by JD that captured significant patterns within the data and were relevant to the research aim.
4. *Developing and reviewing themes.* This involved checking whether the initial themes accurately represented the data and if they needed further adjustment. Each theme was further examined for potential overlap, which was also resolved at this step.
5. *Refining, defining, and naming themes.* The process of refining the themes was dynamic and iterative, with JD, EF, AWL, and VS revisiting them multiple times to ensure clear distinctions. While the authors’ interpretations were largely consistent, ongoing discussions were necessary to address nuances and ensure the analysis accurately reflected participants’ experiences and aligned with the research question.
6. *Writing up.* The final step was to compile and present the findings in a clear and systematic manner. This included writing a detailed analysis of each theme, illustrating them with examples from the data. All authors contributed to this final phase.

All analysis was conducted in Swedish, after which JD translated the themes and illustrative quotes into English. All authors carefully reviewed the translations to ensure that the meaning of the original texts was conveyed as accurately as possible. While translation inevitably introduces interpretive nuances, these steps were taken to mitigate potential misinterpretations and strengthen the credibility of the analysis.

The research team was interdisciplinary, comprising diverse fields such as social anthropology, medicine, sociology, social work, epidemiology, public health, psychology, and nursing. JD, a researcher with a background in social anthropology and experience in conducting qualitative studies with PwMS (34), approached the study with sensitivity to the emotional and relational aspects involved in managing a chronic, and potentially stigmatizing, condition at work. This perspective shaped her attentiveness to how participants navigated disclosure, concealment, and everyday self-management. During coding and theme development, JD—and the rest of the research team interpreted both the practical strategies described and the emotions, meanings, and reasonings underlying them. JD’s previous work on MS disclosure heightened her awareness of related themes. Thus, she continuously kept reflexive notes and revisited the data to consider how her prior assumptions shaped interpretation. Regular team discussions were further central to this reflexive process by challenging each other’s readings, assumptions, and making space for alternative interpretations.

### 2.3 Ethical considerations

The participants were given fictitious names in the transcriptions, along with any mentioned individuals, locations, and workplaces, to protect their identities. In the presentation of the findings, all quotes are anonymized, with minor changes adapted (in case needed) to protect the anonymity of the participants, without changing the content of the quote. All data has been kept in accordance with data protection regulations. Ethical approval was received from The Swedish Ethical Review Authority (Registration number: 2020-04996).

## 3 Results

The 16 participants’ MS experience varied in duration, spanning from 6 months to 18 years since receiving their diagnoses, and their ages ranged from 25 to 57 years. The majority (*n* = 12) were employed full-time, and they represented a wide range of professions. Many of them, however, held office-based jobs (*n* = 10). There was a notable presence of managerial roles (*n* = 5), suggesting varied levels of responsibility. Moreover, disclosure statuses at work varied among the participants, ranging from fully disclosed (*n* = 6), to partially disclosed (*n* = 6), to fully concealed (*n* = 4).

**Figure 1:**
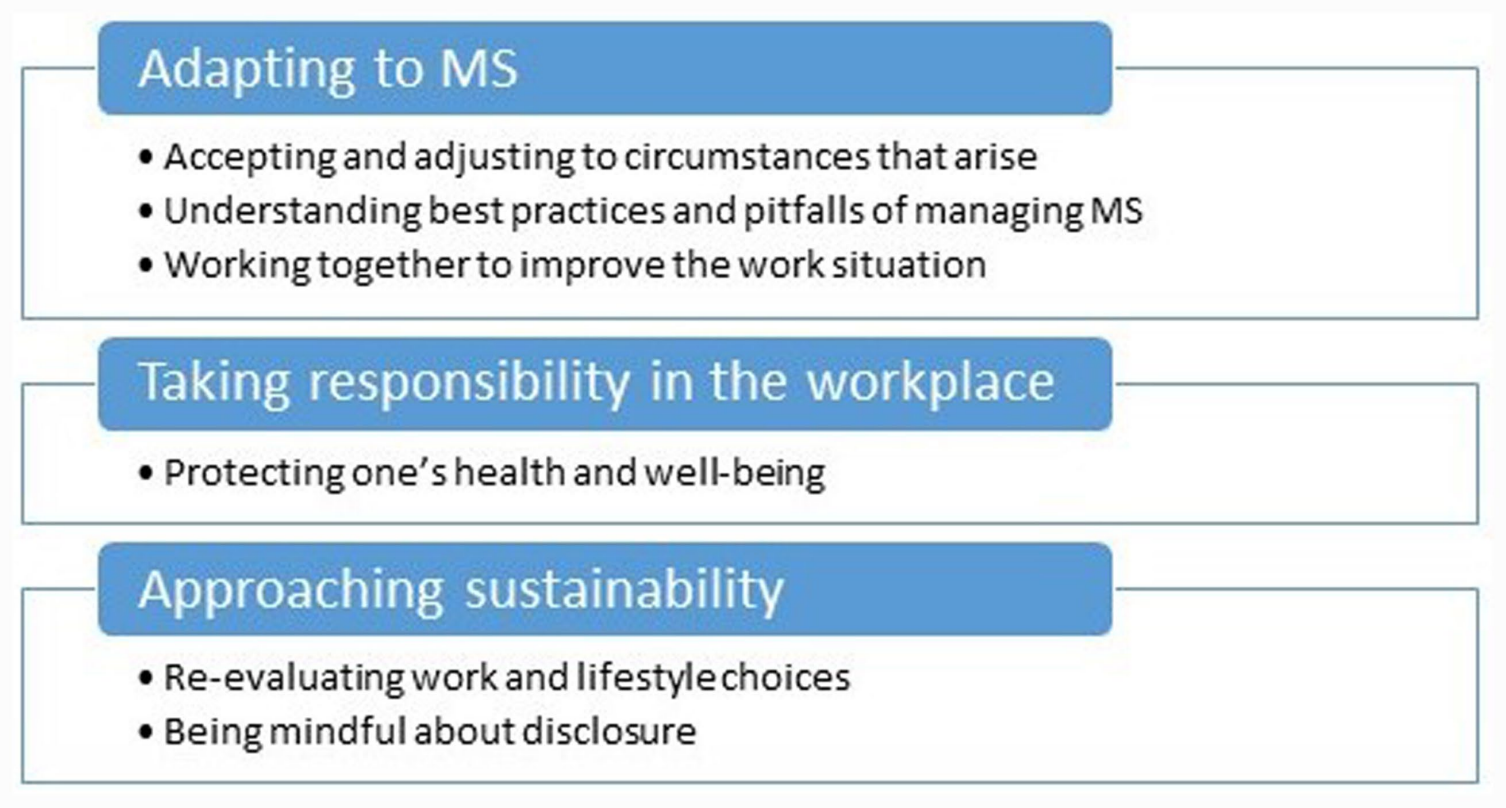
Overview of themes.

In the analysis of the interviews, three main themes and six sub-themes were identified (see Figure 1), which are described below.

### 3.1 Theme: adapting to MS

This theme highlights the mental and practical adjustments necessary for managing MS in the workplace. It shows how critical reflection on past approaches marked a turning point for some participants, where strategies that were more in line with MS needs started to emerge. Deep-rooted habits, however, made it difficult not to fall back into old patterns, showing how adjusting oneself to a chronic condition is not only something that occurs at disease onset, but is a continuous effort.

#### 3.1.1 Accepting and adjusting to circumstances that arise

As described by the participants, learning to live with MS often required changes in their daily lives and mindset which could be challenging, especially for those used to high levels of productivity. Some expressed difficulties in mentally adapting to MS, and their responses reflected a conflict between looking after their health and being passionate about their work. This conflict did not just happen at the beginning, but was something that participants continuously faced and found challenging.

*“I cannot be at the front of the battle line [anymore]. But it’s very, very hard mentally because I’m such a Type A personality, over-achieving, over-ambitious… And then to sort of accept, to allow yourself not to be that person […], that’s hard. All my life, I have identified myself very much with my work […]. Because my work has been 80 % of me.”* (Tobias, 57 years old, time since diagnosis: 8 years)

Many described a process of letting go of old routines and adapting to new circumstances. For some, this meant choosing an approach that felt unfamiliar in the moment but was understood as beneficial in the long term.

*“That’s yet another strategy, I realize now. Like, being in a context, but demanding nothing from myself […]. Not demanding that I have to drive the conversation forward or initiate contact… But is not it fun just to see what happens? […] Just sit down and take it easy and lower the demands [of myself] in the context.”* (Klara, 39 years old, time since diagnosis: 2 years)

The participants acknowledged that their own behavior had sometimes contributed to an overwhelming situation, showing how critical reflection was a first step in knowing how to approach MS differently. By looking back, some participants shared how they wished they had been kinder to themselves during the initial stages of adapting to MS, rather than trying to carry on as normal.

However, part of adapting to MS was not just about challenging oneself and breaking old habits; it was also about drawing on existing skills and personal strengths. For example, being proactive and solution-oriented were two attributes commonly highlighted as contributing to better outcomes.

#### 3.1.2 Understanding best practices and pitfalls of managing MS

Managing MS was a constant balancing act in the participants’ lives, where they had to navigate symptoms and other manifestations of the disease while striving to remain productive at work. Becoming aware of and learning how to avoid stress or exhaustion was frequently mentioned, as was learning how to regulate themselves better and make the most effective use of their limited capacity. As described, the better they understood their disease and how it “behaved,” the more adept they were at recognizing early warning signs. Participants who had lived with MS for a long time shared how they had gradually learned to manage their condition, and by that, achieving a sense of empowerment.

*“Actually, I was in a much worse shape twenty years ago than I am now, so I would say that I’m in better… I’m in control of my MS. I know how it works. I know what I can do, I know what I cannot do […]. And that is what has become my successful concept.”* (Louise, 41 years old, time since diagnosis: 18 years)

Given the fluctuating nature of MS, the participants often discussed the importance of proactive planning and creating good conditions for themselves. They knew that they might have days where their symptoms are manageable, allowing them to work effectively, while other days could be significantly more challenging. Furthermore, participants described how they adapted their strategies based on the specific symptoms or limitations they faced. For instance, those with fatigue described how they withdrew socially to save energy, while those with mobility issues planned shorter routes to and from work to minimize physical strain. Participants also reported experiencing more stable periods, allowing them to work with little or no discomfort. However, the strategy in these cases was to remain attentive as they knew a stable period could end abruptly.

#### 3.1.3 Working together to improve the work situation

In addition to their own strategies, the importance of being acknowledged by managers and/or colleagues during difficult moments was further emphasized by some participants. It was, for example, shared how honest dialogues with one’s managers or co-workers could make one realize a work change was necessary.

*“It did not suit me this jumping from early morning to afternoon and well into the night and then shifting the day back and forth, week after week, after week. It worked for a while but no, I could not take it, it was too hard… It was the person in charge in that department where I am who noticed that I did not look well, asked about it and then I said that ‘no I cannot stand this jumping back and forth,’ then they asked if I wanted to go back [to work] day-time only (…) and it could be changed immediately without any problems.”* (Emil, 35 years old, time since diagnosis: 2 years)

Some participants also described seeking help from MS-focused rehabilitation programs, and that they continuously tried to transfer these learnings into their daily work life. For instance, how to slow down at work well before one’s energy levels are at their lowest.

### 3.2 Theme: taking responsibility in the workplace

This theme highlights the strong sense of responsibility participants expressed regarding managing their MS in the workplace. While this sense of responsibility empowered some, it also demanded constant attention and effort. It further shows how the participants actively sought to create conditions that were more conducive to their health, using the resources available to them in the workplace. Lastly, the theme illustrates the challenge of balancing health needs with work demands, and how the work context could both facilitate and hinder self-management.

#### 3.2.1 Protecting one’s health and well-being

It was evident that personal responsibility was at the core of their strategies. Participants emphasized that, due to having much narrower health margins compared to those without chronic illness, they felt an even greater need to carefully manage their health, as they understood that failing to maintain their routines could lead to setbacks or long-term consequences. Some noted that when they compromised on or deviated from their routines, they experienced a noticeable decline in their overall well-being, which in turn affected their work performance. Thus, they were determined to prioritize what was necessary to manage their MS effectively.

*“I can notice that over the years, the way the disease is noticeable is that I am worse at handling stress. Fatigue kicks in a little, so I have to make sure I fend that off, and I kind of have to go to bed on time. I must make sure that I can rest.”* (Malin, 49 years old, time since diagnosis: 11 years)

The participants further discussed the importance of being assertive about health needs, having the courage to make demands regarding their work setting, and having clear communication with managers and colleagues to create an environment more favorable to them. For some participants, this entailed disclosing the diagnosis. Others, however, chose not to share their health condition but still communicated their needs, focusing on practical solutions. Regardless of the approach, focus remained on creating work conditions that supported their health. However, participants described that it sometimes took time to learn how to effectively address health needs, as this involved not only understanding what they needed but also presenting them in a way that would foster understanding from their managers and colleagues. Participants also expressed that they sometimes needed to set firmer boundaries with others in the workplace, to make sure they did not take on more work than they could handle.

However, taking a stance for their health also involved using subtler, less visible strategies. For example, participants described managing their symptoms without informing their managers or colleagues. This could include occasionally resting during the workday and resuming tasks in the evening when feeling more energetic. Such maneuvers allowed participants to better balance their health needs with their professional responsibilities. For some, however, this was also an expression of how far they were willing to go to hide their difficulties with MS, and to avoid letting MS become a known fact.

Many of these subtler strategies for managing MS were facilitated by making use of already established workplace benefits. For some participants, flexibility was not only beneficial but also a decisive factor in choosing a particular workplace or deciding to stay. For example, several participants were employed in workplaces that offered flexible working arrangements and the option to work from home. These benefits were not implemented for employees with MS specifically, but were intended to benefit all staff members. By using them, participants could adapt their work environment and schedules to better accommodate their health needs. Flexible hours enabled the participants to manage fatigue and other symptoms more effectively by adjusting work schedules according to their energy levels. It was further described how not having to commute to and from work every day meant they could spend time working more efficiently, getting more work done at home in a more comfortable and calmer environment. This further allowed them to regain balance, especially during challenging periods related to their MS, while avoiding the pressure of maintaining a façade when they were not feeling well emotionally.

It was evident that actively looking after themselves fostered a sense of accomplishment and empowerment among the participants, which motivated them to continue their efforts. However, this responsibility could also be demanding as it required consistency, attentiveness, and a disciplined approach to routines. For instance, participants explained how, despite still being able to perform tasks, they had to forgo other, more social, aspects of their worklife in order not to exacerbate their condition.

*“I choose not to participate in all the stuff because it becomes too exhausting, and sometimes I might have to go home and rest, but above all, when it’s a lot of walking or different types of physical activities that I have not done before, and I do not know how my body will respond. If I do not know how it will respond, I say no.”* (Elisabet, 29 years old, time since diagnosis: 12 years)

The participants further discussed how the broader work context could either support or pose obstacles to their ability to manage MS. Examples were provided of both healthy work cultures that foster shared responsibility to make things work, and dysfunctional cultures where the burden of responsibility tends to fall more heavily on the individual.

However, some participants reported instances where their diagnosis was overlooked despite being in a supportive work environment and having communicated their MS limitations. This and similar instances led some to be realistic about their expectations of others.

*“The insight that it’s actually me who must take responsibility for taking care of myself (…). There is no one else who speaks up [for me], and there is no one else who says ‘oh it hurts.’ No one says ‘the light, it’s too bright.’”* (Klara, 39 years old, time since diagnosis: 2 years)

It was often understood that waiting for others to accommodate their needs was not an option, but that it was ultimately their own responsibility to ensure that these were met.

### 3.3 Theme: approaching sustainability

This theme captures how PwMS responded not only to their immediate health needs in striving for sustainability but also to uncertainty about their future capabilities and the potential implications for their life trajectory. While participants described practical strategies, such as exploring new career options, scaling down responsibilities, and choosing roles that better suited their health, their decisions were guided by a broader effort to maintain stability over time. Finally, the theme explores how sustainability guided the participants in how they chose to approach the issue of disclosure at work.

#### 3.3.1 Re-evaluating work and lifestyle choices

MS prompted many participants to review their work life and how they want to spend their time. For some participants, this meant exploring an alternative career path. This was sometimes necessary as worsening symptoms did not allow them to continue with the same type of work as before, as they could potentially place themselves or others at risk. In other cases, it came from a deeper reflection on their work life and the long-term impact imposed by MS, and a wish to be proactive with lifestyle choices.

Work was often referred to as an important component in the participants’ lives. Consequently, choosing a new career was rarely a rushed, but rather a carefully thought-out decision. For example, participants found it necessary to alter the type of employment or reduce responsibilities to better manage the interplay between work life and MS. However, while the focus most often centered around adapting one’s work and career to accommodate the challenges of MS, participants also stressed the importance of not entirely abandoning their professional aspirations due to the disease. This involved steering one’s career towards a sustainable path, yet still aligned with professional interests, showing efforts to balance necessary adaptation while protecting valued aspects of their work.

*“I’m proud of it, I’ve made sure to get myself to the right [work]place. I do something I find fun, I get paid well, and it works with my diagnosis.”* (Elisabet, 29 years, time since diagnosis: 12 years)

#### 3.3.2 Being mindful about disclosure

In terms of disclosing or concealing MS at work, participants described that they were often mindful of how much personal information to share and the manner in which they shared it, considering aspects such as timing, outcomes, and their careers, reflecting a continuous negotiation of relational dynamics, as well as strategic, practical, and emotional considerations in the workplace.

Regarding timing of disclosure, they recognized that it involved not only deciding when to share their diagnosis but also creating conditions for a supportive response. Furthermore, disclosure was an ongoing process, not a one-time event. Starting a new job or interacting with new managers and colleagues raised a recurring question of when and how to disclose one’s condition.

They further discussed the importance of preparation when initiating discussions about MS at work. For example, participants described situations where, without adequate preparation, they sometimes shared more information than they were comfortable with. By thinking ahead and planning their responses, they were able to manage these discussions more effectively. This highlights the careful planning involved in managing how MS is perceived by others, not only to protect privacy but also to maintain normality and control in everyday work life.

The participants did not always disclose MS per se, but focused health-related discussions on symptoms. By choosing what pieces of information they felt comfortable sharing, they could redirect focus from the diagnosis towards practical solutions and adjustments. Martin described: *“I did not tell a long story about my background when I started my job, but I explained that my eyesight was bad.”* (Martin, 40 years old, time since diagnosis: 16 years).

Whether or not to disclose MS was also influenced by work performance and stability. Having an established and secure position in one’s job was further shared as a factor facilitating disclosure.

Other participants expressed that, despite managing their condition well, they worried that their manager or colleagues might still have doubts or negative assumptions about their ability to perform. Some felt more motivated to share their diagnosis once they could prove their ability to work effectively despite their MS. Conversely, some chose to conceal their diagnosis when their MS did not significantly impact their job performance, arguing that disclosure was not necessary.

The participants further stressed the importance of taking a long-term perspective and considering the potential implications of disclosing the diagnosis. This involved looking beyond the immediate challenges or crises one might face here and now. It was noted that rushed or impulsive decisions about disclosure could lead to unintended consequences or regret. One key point highlighted was the irreversibility of disclosure. Most did not see it as advisable to disclose MS to everyone in the workplace. Instead, they thought it was wiser to identify who genuinely needs to know, such as one’s closest manager or colleagues. While many stressed the importance of weighing the potential consequences of disclosure, it was also seen as vital to challenge any exaggerated fears or concerns. Some suggested de-dramatizing the act of disclosure, as others at work may not view one’s health situation as negatively as expected.

## 4 Discussion

This qualitative study explored the experiences of PwMS as they manage MS in the workplace, focusing on the strategies they employ to facilitate and navigate their work lives.

The PwMS employed various strategies, concentrating largely on adapting to MS mentally as well as practically, taking an active stance for their health, and making choices based not only on their current health status but also on potential future circumstances. These findings align with existing literature, highlighting the central role PwMS play in managing their own condition (26, 35).

It was further observed that adapting to MS sometimes required considerable mental adjustment and self-learning through trial and error. Similar findings have been reported in a qualitative study by Ploughman et al., which, while not focusing specifically on individuals’ workplace experiences, highlighted the complexity of self-management and the challenges faced by PwMS in seeking solutions to manage MS (36). In the present study, we found that while the experience was challenging for some, it was often through this trial-and-error process that more effective and kinder approaches to managing MS eventually could emerge. Quantitative evidence from Honan et al. (37) reinforces the observation of mental adjustments, showing that both perceived cognitive difficulties and objective cognitive test performance are significant predictors of employment outcomes in PwMS. This underscores that cognitive challenges, real or perceived, shape work sustainability and require continuous internal adjustments.

Our findings illustrated, however, that managing MS is not solely an individual responsibility or shaped by personal strategies; rather, it is influenced, co-constructed, and constrained by the specific conditions of the work environment. Participants’ efforts could be either facilitated or hindered depending on the type and level of support available in their environment. They described taking responsibility for their health as empowering yet demanding, especially when navigating workplace challenges. From a critical perspective, while this heightened sense of personal agency can be understood as a natural response to coping with a chronic condition, it also places an excessive burden on individuals and reinforces neoliberal expectations that workers must adapt to organisational norms. This individualization of responsibility may be reproduced, intentionally or not, by managers and other workplace actors. Previous research (38–40) shows that although employers acknowledge having a role in supporting workers with chronic conditions, they often view health management as the employee’s responsibility, with support largely depending on disclosure and the employee–manager relationship. Structural and relational barriers, such as stigma, high work demands, and reluctance to provide accommodations, can significantly hamper employees’ agency (40).

A recurrent theme in the participants’ descriptions was not only the need to adapt their work lives to accommodate MS, but also having the opportunity or the right conditions to regain balance and give themselves enough time to recover. For many, this was made possible by the flexibility of their work arrangements. It was further demonstrated that managing a chronic disease at work is not just about visible or vocal actions. Often, it involves a quiet, personal effort that may go largely or completely unnoticed by others in the workplace. Strauss and colleagues’ concept of illness work (21, 22), referring to the extensive yet often invisible tasks individuals perform to maintain health and functionality, such as monitoring symptoms, planning energy, and adapting routines, provides a useful lens for understanding these efforts involved in managing MS at work. Many of our participants’ strategies occurred “behind the scenes”; in their mindset, consistency in routines, continual attention to their condition, and countless small decisions made each day to improve their ability to manage the disease effectively. These “covert” (41) strategies represent key components of illness work and contrast with “overt” (41) strategies, such as disclosing a diagnosis or requesting accommodations, which are more visible to the environment and socially acknowledged. While PwMS often employ both types of self-management, overt efforts typically receive the most recognition, as they are more tangible. Greater attention is needed, however, for covert strategies, as they constitute a hidden labor that is critical for sustained work participation but often goes unrecognized. For workplace support to be fully effective, it must address not only visible requests but also create conditions that enable employees to manage the invisible, yet significant, covert self-management strategies that underpin their ability to participate fully in work.

Previous studies on PwMS highlight the importance of personalized support for sustainable work participation, through attention to individuals’ specific needs (42, 43). Our participants utilized self-monitoring, trial-and-error, and critical self-reflection to manage their condition while meeting work demands. While empowering, this self-initiated responsibility may indicate a lack of formalized processes, placing responsibility largely on the individual and shifting attention away from environmental factors. Further, our findings underscore that personalized support should be treated a continuous, context-dependent process to facilitate sustainable work participation.

Furthermore, our findings echo Bury’s concept of biographical disruption (23), showing how living with and adjusting to MS unsettled participants’ taken-for-granted habits, prompting them to rethink behavioral patterns and, in that sense, reconstruct their biographies. In addition, the theme “Approaching sustainability,” which involved reassessing career plans and choosing roles aligned with capabilities, can, following Bury, be seen as a way to restore coherence in one’s life trajectory despite MS. This highlights that self-management involves not only coping with immediate health needs but also attending to long-term implications for health, identity, and life planning.

Moreover, the findings revealed the diverse ways in which the participants chose to communicate about MS at work. A majority reported that they had disclosed MS in some capacity. Participants navigated the complexities of disclosing or concealing their MS by carefully evaluating the timing, potential reactions, and impact on their professional lives. A notable pattern of partial disclosure emerged among the participants, who shared only specific aspects of their MS or disclosed their diagnosis to certain colleagues while keeping it hidden from others. This approach allowed them to maintain privacy and avoid feeling overly exposed. These findings suggest that partial disclosure may be a more effective strategy than full disclosure for managing workplace perceptions and impacts, depending on individual preferences and circumstances. To the best of our knowledge, this study is the first to report this nuanced approach to disclosure among PwMS in a workplace setting. The concept of partial disclosure (44) fits within emerging literature on disclosure and concealment, which emphasizes that sharing health information is not a binary act but an ongoing, revisited process (30). From this perspective, disclosure and concealment are not opposite states but exist on a continuum. Partial disclosure may serve functions similar to concealment, such as managing anticipated stigma and protecting identity (45), while still allowing individuals to access necessary workplace support. Viewing partial disclosure through this lens highlights its role as an intermediate strategy within the evolving context of living and working with MS.

### 4.1 Methodological considerations

#### 4.1.1 Strengths and limitations

The rich data of the perspectives of PwMS represent the main strength of the current study. Our study offers a nuanced account of MS strategies in the workplace; not only focusing on these efforts as such, but also emphasizing the processes through which they emerge. Our results are, therefore, a valuable contribution to the limited research available on these topics. Using data from 16 participants, representing different ages, sexes, disease durations, and occupational categories, further enhances the diversity and depth of the findings. Credibility was enhanced by using a semi-structured interview guide with all participants. This ensured that each participant had the same opportunity to respond to the questions, which led to consistency in the data collection process.

An additional strength was the diverse educational and professional backgrounds of the authors. This contributed to a richer and more holistic understanding of the study and helped raise critical questions that improved the interpretation of the findings. Moreover, most of the authors were involved in the analysis. This helped verify and validate the findings and added robustness to the study, as it enhanced the trustworthiness of the results.

Moreover, extending the “illness work” and “biographical disruption” frameworks into the workplace represents a methodological strength. Together, these perspectives allow for a more nuanced understanding of self-management. While “illness work” (21, 22) captures the everyday practical efforts involved in managing a chronic condition, “biographical disruption” (23) situates these efforts within broader considerations of identity and life trajectory.

Providing descriptions of the research context and participants made it possible for others to assess the potential transferability of the findings in other research settings. It is important to note, however, that the Swedish context is distinctive due to its social insurance and employment laws (31), offering specific protections and support to PwMS, as well as other patient groups, conditions that may not be available in other countries. These policies can significantly influence the likelihood of disclosure among PwMS in Sweden, making our findings more relevant for countries with similar systems. Moreover, one could have considered complementing the interviews with a standardized measure of work-related difficulties, such as the MSWDQ-23 (46), to inform comparisons and transferability across populations.

Secondly, relying on video and telephone interviews may have affected the depth and nuance of the data collected, as physical presence often allows for better observation of non-verbal cues and a more natural rapport. However, studies suggest that video and telephone interviews offer several advantages, particularly in terms of participants’ energy and emotions (47). As in other studies, we found that this approach increased accessibility and convenience (48) for our participants. It also made it possible to include participants from various geographical locations without the need for travel, reducing barriers for those with mobility issues or time constraints. Furthermore, this approach created a more comfortable and flexible environment, potentially leading to more open and honest responses.

Moreover, this study largely reflects the experiences of office-based workers, which likely influenced the strategies they were able to employ for managing MS in the workplace. Office settings generally provide greater opportunities for rest and flexibility, opportunities that may be far less available in physically demanding jobs. Therefore, the transferability of our results is likely limited to similar sedentary, office-based work environments. Different approaches may be necessary for those in more physically demanding jobs, potentially involving more physical accommodations and job modifications (49, 50). Hence, further research should explore how PwMS in a wider range of work environments manage their condition.

## 5 Conclusion

This study provides insights into the experiences of those living and working with MS. The results demonstrate the active agency in managing their condition through a continuous “trial and error” process. While this active agency empowered PwMS, it also placed significant responsibility on them, which could sometimes lead to a demanding experience, highlighting the challenges of managing a chronic condition alongside work life. Moreover, while our study demonstrates that individuals’ own efforts to take responsibility for their health at work are crucial, they represent one part of a larger system. Our findings also show the importance of flexibility and control in the workplace, as well as a responsive network and a supportive work culture in providing individuals with conditions that facilitate more effective self-management. Employers and policymakers should consider these factors when designing workplace support systems for PwMS, ensuring that structures and practices are supportive and flexible so employees with chronic conditions, such as MS, can manage their health while meeting work responsibilities.

## Data Availability

The data that support the findings of this study are available on request from the corresponding author. The data are not publicly available due to ethical restrictions. Requests to access the datasets should be directed to EF, emilie.friberg@ki.se.
